# Glaucoma Alters the Circadian Timing System

**DOI:** 10.1371/journal.pone.0003931

**Published:** 2008-12-12

**Authors:** Elise Drouyer, Ouria Dkhissi-Benyahya, Christophe Chiquet, Elizabeth WoldeMussie, Guadalupe Ruiz, Larry A. Wheeler, Philippe Denis, Howard M. Cooper

**Affiliations:** 1 Department of Chronobiology, INSERM, U846, Stem Cell and Brain Research Institute, Bron, France; 2 University of Lyon, Lyon I, UMR-S 846, Lyon, France; 3 Centre National de la Recherche Scientifique, Lyon, France; 4 Department of Ophthalmology, CHU de Grenoble, Faculté de Médecine, Université Joseph Fourier, Grenoble, France; 5 Pfizer, Inc, San Diego, California, United States of America; 6 Allergan Inc., Irvine, California, United States of America; 7 Department of Ophthalmology, CHU de Lyon Hopital Edouard Herriot, Lyon, France; Institut de la Vision, France

## Abstract

Glaucoma is a widespread ocular disease and major cause of blindness characterized by progressive, irreversible damage of the optic nerve. Although the degenerative loss of retinal ganglion cells (RGC) and visual deficits associated with glaucoma have been extensively studied, we hypothesize that glaucoma will also lead to alteration of the circadian timing system. Circadian and non-visual responses to light are mediated by a specialized subset of melanopsin expressing RGCs that provide photic input to mammalian endogenous clock in the suprachiasmatic nucleus (SCN). In order to explore the molecular, anatomical and functional consequences of glaucoma we used a rodent model of chronic ocular hypertension, a primary causal factor of the pathology. Quantitative analysis of retinal projections using sensitive anterograde tracing demonstrates a significant reduction (∼50–70%) of RGC axon terminals in all visual and non-visual structures and notably in the SCN. The capacity of glaucomatous rats to entrain to light was challenged by exposure to successive shifts of the light dark (LD) cycle associated with step-wise decreases in light intensity. Although glaucomatous rats are able to entrain their locomotor activity to the LD cycle at all light levels, they require more time to re-adjust to a shifted LD cycle and show significantly greater variability in activity onsets in comparison with normal rats. Quantitative PCR reveals the novel finding that melanopsin as well as rod and cone opsin mRNAs are significantly reduced in glaucomatous retinas. Our findings demonstrate that glaucoma impacts on all these aspects of the circadian timing system. In light of these results, the classical view of glaucoma as pathology unique to the visual system should be extended to include anatomical and functional alterations of the circadian timing system.

## Introduction

The hallmark of glaucoma is the degenerative loss of retinal ganglion cells (RGCs) and their optic nerve fibers. In the absence of adequate treatment glaucoma inevitably leads to blindness and is expected to affect more than 60 million people worldwide by 2010 [1]. Although glaucoma results from multiple factors and likely comprises a family of diseases, raised chronic intraocular pressure (IOP) represents a significant risk factor [2]–[7]. The primary symptom of glaucoma is an initial reduction of the peripheral visual field with the degree of loss evolving proportional to RGC loss [8]–[9]. Previous studies in both humans and in monkey models of glaucoma initially reported that larger soma sized RGCs were primarily susceptible to injury or damage [10]–[13]. However, a recent study found that all classes of RGCs follow a similar time course of degeneration beginning from the early stages of the disease [14].

In the mammalian retina, the light sensitive RGCs that express the photopigment melanopsin [15]–[18] comprise a specific subset (<1%) of large sized ganglion cells that regulate the photic synchronisation of circadian rhythms and more generally, a spectrum of “non-visual responses” to light including the acute suppression of melatonin, pupillary constriction, alertness and masking [19]–[23]. These RGCs mainly project to “non image-forming” structures including the suprachiasmatic nucleus (SCN), intergeniculate leaflet (IGL), olivary pretectal nucleus (OPN), lateral hypothalamus and preoptic regions [16], [24]–[25]. Minor input has also been demonstrated to the dorsal lateral geniculate nucleus (dLGN [18]) and superior colliculus (SC [24]). Functional invalidation of melanopsin photopigment in *Opn_4_* ^−/−^ mice leads to attenuated circadian [16], [20], [21], [26], pupillary [27] and electrophysiological responses to light [28] while the absence of functional rods, cones and melanopsin results in a total inability to respond to light [21], [26]. Ocular pathologies and blindness in humans are also associated with circadian and sleep disorders that depend on the degree of conserved light perception [29]–[31]. Presently, however, there is no clear support for the commonly held hypothesis of the impact of glaucoma on circadian and/or sleep disturbances and the degenerative loss of melanopsin RGCs in this pathology is controversial. One study [14] provided evidence that melanopsin RGC degeneration is proportional to that of the entire ganglion cell population, while a second report [32] claimed selective sparing of melanopsin neurons.

To explore the hypothesis that glaucoma leads to alterations in both the visual and non-visual systems we used a rat model of glaucoma with laser-induced chronically elevated IOP [33] in a strategy that combines three novel functional and behavioral approaches. We address several questions that have not previously been probed in studies of glaucoma. Based on findings that RGC somas degenerate in glaucoma, to what extent are their axonal fiber projections to different brain target structures affected? If the photic input pathway to the SCN is altered in glaucoma, does this impact on the capacity for light entrainment by the circadian timing system? How do degenerative changes of glaucoma alter photopigment expression in the inner and outer retina?

## Materials and Methods

### Animals

Male Wistar rats (n = 30) were housed individually in propylene cages, under a 12:12 LD cycle, with food and water *ad libitum*. Eye surgery for inducing experimental glaucoma was performed at 3–6 months of age. Anatomical and behavioral investigations were undertaken on animals between the ages of 6–12 months. Experiments were conducted in accordance with current national (Décret No. 87-848), international (EU guidelines) and institutional regulations for animal husbandry and surgical procedures.

### Laser Technique for raised intra-ocular pressure

**Figure 1:**
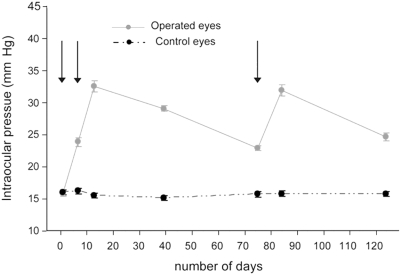
Levels of intraocular pressure (IOP) of rats following treatment with Argon laser photocoagulation. IOP in the operated eyes are compared with that in the unoperated eyes from the same individuals (n = 12). Arrows represent the times of the three laser surgeries. The serial sequence of laser treatments assures a long-term chronic elevation of IOP.

Argon laser treatment (blue-green argon laser; Coherent, Palo Alto, CA) of the episcleral veins (responsible for aqueous outflow tissue) was used to induce chronic elevation of IOP according to previous methods [33]. Rats were anesthetized with a mixture of ketamine (50 mg/kg), acepromazine (1 mg/kg), and xylazine (25 mg/kg). Laser treatment was performed unilaterally (n = 12, for the anatomical study) or bilaterally (n = 5, for the behavioral study) in three stages on the episcleral veins within 0.5 to 0.8 mm from the limbus and on the veins in the limbus. The first two surgeries occurred one week apart and the third was performed 2 months after the second one. The amount of energy used was 1W for 0.2 seconds, delivering a total of 230 to 550 spots (50–100 mm spot size) with the laser treatments. IOP was monitored using a tonometer (Tono-Pen; Mentor, Norwell, MA). Figure 1 illustrates the progression of IOP in the 12 rats with monocular laser treatments measured from the operated and unoperated eyes. In this model, retinal ganglion cell (RGC) loss was previously demonstrated to be roughly 33% after 3 weeks of elevated IOP.

### Anterograde tracing of retinal projections, CTb immunocytochemistry

#### Injection of Cholera toxin fluorochrome conjugates for qualitative study

Rats with monocular laser surgery (n = 4 of the 12 rats described in laser treatment) were anesthetized with a mixture of ketamine (50 mg/kg) and xylazine (25 mg/kg). Eyes were additionally anesthetized locally using topical application of oxybuprocaïne chlorhydrate. Two rats received a 0.5 mg intraocular injection (6 µl) of Cholera toxin subunit b (CTb) Alexa Fluor 594 conjugate (red fluorescence, #C-22842, Molecular Probes, CA) in the operated right eye and a 0.5 mg intraocular injection (6 µl) of CTb Alexa Fluor 488 conjugate (green fluorescence, #C-22841, Molecular Probes, CA) in the unoperated (control) left eye. The 2 other rats received the same two intraocular injections of CTb fluorochromes but with the reverse Alexa Fluor 488 conjugate in the operated right eye and Alexa Fluor 594 conjugate in the control left eye.

#### Injection of Cholera toxin for quantitative analysis

Rats with monocular laser surgery (right eye, n = 8 of the 12 rats described in laser treatment) and controls with no laser surgery (n = 8) were anesthetized with a mixture of ketamine (50 mg/kg) and xylazine (25 mg/kg). Eyes were additionally anesthetized locally using topical application of oxybuprocaïne chlorhydrate. Rats with monocular induced glaucoma received a 0.5 mg injection (6 µl) of CTb (#130A, List Biological Laboratories Inc) in the operated eye. Control rats with no laser surgery received the same injection in their right eye.

#### Treatment of Brain Sections and CTb Immunocytochemistry

For both the CTb fluorescence and diaminobenzidine (DAB) studies, 48 hr after the injection, all animals were deeply anesthetized with a lethal intraperitoneal injection of sodium pentobarbital (150 mg/kg) and perfused intracardially with warm (37°C) heparinized saline followed by 300 ml of Zamboni's fixative at 4°C. Brains were removed, and post-fixed overnight in a mixture containing the same fixative with 30% sucrose for cryoprotection at 4°C. Serial coronal sections were made at 50 µm on a freezing microtome and all brain sections were collected. Sections from the animals injected with the CTb anterograde tracer coupled to a fluorochrome were directly mounted on slides, dehydrated and coverslipped.

All sections from all animals injected with CTb were processed at the same time to obtain identical levels of tissue staining for data analysis. Endogenous peroxidase was first suppressed using a solution of 50% ethanol in saline with 0.03% H_2_O_2_. Free-floating sections were rinsed briefly in PBS (0.01 M, pH 7.2) containing 0.3% Triton and blocked with 1% bovine serum albumin. Sections were incubated in the anti-CTb primary antibody (dilution 1∶3,000) for 3 days at 4°C. Immunoreactivity was visualized using a Vectastain ABC Elite kit (PK-6100, Vector Laboratories, Burlingame, CA), followed by incubation in 0.2% 3,3′- DAB with 0.5% ammonium nickel sulfate and 0.003% H_2_O_2_ in Tris buffer (0.05 M, pH 7.6). Sections were then mounted on slides, dehydrated and coverslipped.

### Quantification of retinal projections

Retinal projections to the brain were quantified on sections processed for DAB immunocytochemistry, based on a published methodology [34] that has been applied in several previous studies of brain organization [35]–[37]. Briefly, quantitative levels of the optically dense DAB immunolabel product were measured using computer-assisted image analysis (Biocom, Les Ulis, France). Optical density of label was measured bilaterally in each structure from digitized images. Quantification of label is determined from the total integral optical density of labeling. Integral density takes into account both the surface area and pixels density. The integral optical density was obtained by first subtracting the background density value. Optical density was measured from all the sections of each structure that receives RGC fiber projections except for the superior colliculus (1 of 2 sections).

### Analysis of Retinal Opsins

At the end of the behavioral study the two retinas were pooled from each of the animals (n = 5 binocular induced glaucoma rats and n = 5 control rats, age 14–16 months). Retinas were collected from the animals between ZT8-ZT9, after the animals had been re-entrained to a 100 lux LD cycle. Total RNA was extracted using GenEluteTM Mammalian Total RNA Miniprep Kit (Sigma) according to the manufacturer's instructions and subsequently subjected to DNase digestion. Total RNAs was reverse transcribed using random primers and MMLV Reverse Transcriptase (Invitrogen). Real-time PCR was then performed on a LightCycler™ system (Roche Diagnostics) using the light Cycler-DNA Master SYBR Green I mix. The efficiency and the specificity of the amplification were controlled by generating standard curves and carrying out melting curves and agarose gels of the amplicons respectively. Relative transcript levels of each gene were calculated using the second derivative maximum values from the linear regression of cycle number versus log concentration of the amplified gene. Amplification of the non cyclic control gene 36B4 was used for normalization. Each reaction was performed in duplicate. Primer sequences were the following:

SW opsin forward 5′ -GTACCACATTGCTCCCGTCT-3′
SW opsin reverse 5′ -AGACCTGCTACAGAGCCCAA-3′
MW opsin forward 5′, -GCCTTATGGCCTGAAGACATC-3′
MW opsin reverse 5′-CTGTTGCTTTGCCACTGCTC-3′
Rhodopsin forward 5′-GCAGTGTTCATGTGGGATTG-3′
Rhodopsin reverse 5′-CTGCCTTCTGAGTGGTAGCC-3′
Melanopsin forward 5′-ATCTGGTGATCACACGTCCA-3′
Melanopsin reverse 5′-TAGTCCCAGGAGCAGGATGT-3′
Thy1 forward 5′-CAGGACGGAGCTATTGGCACCAT-3′
Thy1 reverse 5′-ACGGCAGTCCAGTCGAAGGTTCT-3′
PACAP forward 5′-ACAGCGTCTCCTGTTCACCT-3′
PACAP reverse 5′-CCTGTCGGCTGGGTAGTAAA-3′
36B4 forward 5′-GCTCCAAGCAGATGCAGCAG-3′
36B4 reverse 5′-CAGCTGGCACCTTATTGGCC -3′.

### Entrainment of circadian locomotor activity rhythms

For monitoring locomotor activity, rats operated bilaterally to induce experimental glaucoma were used. A total of 10 rats (n = 5 with binocular raised IOP, n = 5 controls) were housed individually in cages equipped with passive infrared motion captors placed over the cages and a computerized data acquisition system (CAMS, Circadian Activity Monitoring System, INSERM, France). Rats were initially maintained for 26 days under a 12:12 LD cycle with broad-band white light (100 lux). Animals subsequently underwent a 6 hr phase delay of the LD cycle, associated with successive decreases of light intensity (from 100 to 10 lux and subsequently from 10 to 1 lux).

Activity records were analyzed with the Clocklab software package (Actimetrics, Evanston, IL). The time of locomotor activity onsets was determined using the Clocklab onset fit algorithm. Animals were considered to be entrained when the fit of the least squares regression line of the activity onsets was stable in relation to lights-off for at least 7 days of in LD. The phase angle, defined as the time difference between the onset of the activity rhythm and time of lights-off was determined for each animal (see [38]).

### Statistical Analysis

Data were analyzed using the SigmaStat software (Systat Software Inc., Point Richmond, CA). The *Shapiro-Wilk W test* was used to test for normality. For comparisons between two groups, parametric t-test was used when data were normally distributed and non-parametric Mann-Whitney test when normality was not achieved. Data is expressed as mean±S.E.M.

## Results

### Consequences of chronic elevated IOP on retinal projections

**Figure 2:**
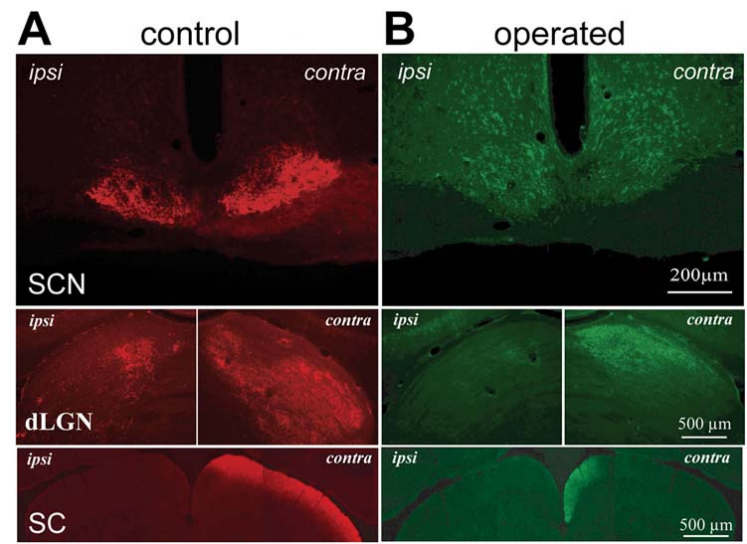
Retinal ganglion cell projections to the ipsilateral and contralateral suprachiasmatic nucleus (SCN), dorsal lateral geniculate nucleus (dLGN) and superior colliculus (SC) from a control and glaucomatous eye. The unoperated control eye was injected with a red fluorescent CTb anterograde tracer (A) and the operated glaucomatous eye with a green fluorescent CTb tracer (B). The images in A and B are taken from the same sections but using different excitation filters. For each of the structures illustrated, the brain hemisphere ipsilateral to the injected eye is to the left and contralateral to the injection is to the right. Thus, the ipsilateral dLGN and SC (red: control) correspond to the same hemisphere as the contralateral dLGN and SC (green: operated). Note that projections to the SCN and SC (montage of 3 photomicrographs) are markedly reduced and that the topographical distribution of the projection in the dLGN is also considerably altered. Part of the material shown is modified from a previous review [69].

All operated eyes of animals treated with laser surgery displayed a consistent chronic increase in IOP (Fig 1). To assess the consequences of chronic hypertension on injury to RGC axonal projections to the brain, intraocular injection of two different fluorescent anterograde tracers were made in the same individual: a green fluorescent tracer in the laser-operated glaucomatous eye and a red fluorescent tracer in the opposite intact eye. Using this coupled, double fluorescence technique, the patterns of retinal projections from the normal and glaucomatous eye can be examined on the same brain section by simply changing the fluorescence excitation filters. Comparison of the retinal fiber innervations (Fig. 2) shows a marked reduction in the density and topographical coverage of the retinal fibers emanating from the glaucomatous eye to all structures examined (SCN, dLGN, SC; see below for IGL ventral lateral geniculate nucleus: vLGN, pretectum: PRT). The loss of retinal innervation from the glaucomatous eye is particularly evident for the SCN where few retinal fibers are present. The dLGN is also affected, with an absence of label in the ventrolateral portion of the contralateral nucleus and a noticeable reduction of the ipsilateral component of the projection. The SC shows a complete loss of fibers in the lateral region. This lateral part of the SC and the ventrolateral part of the dLGN correspond to the temporal and dorsal parts of the visual field [39]–[40]. Although there was variability between individuals in the topographical pattern and degree of reduction of RGC fiber innervation, all 4 cases examined demonstrated a uniform decrease in innervation of the SCN coupled with a patchy distribution or empty regions in the dLGN and SC.

### Quantification of retinal projections to visual and non-visual structures

**Figure 3:**
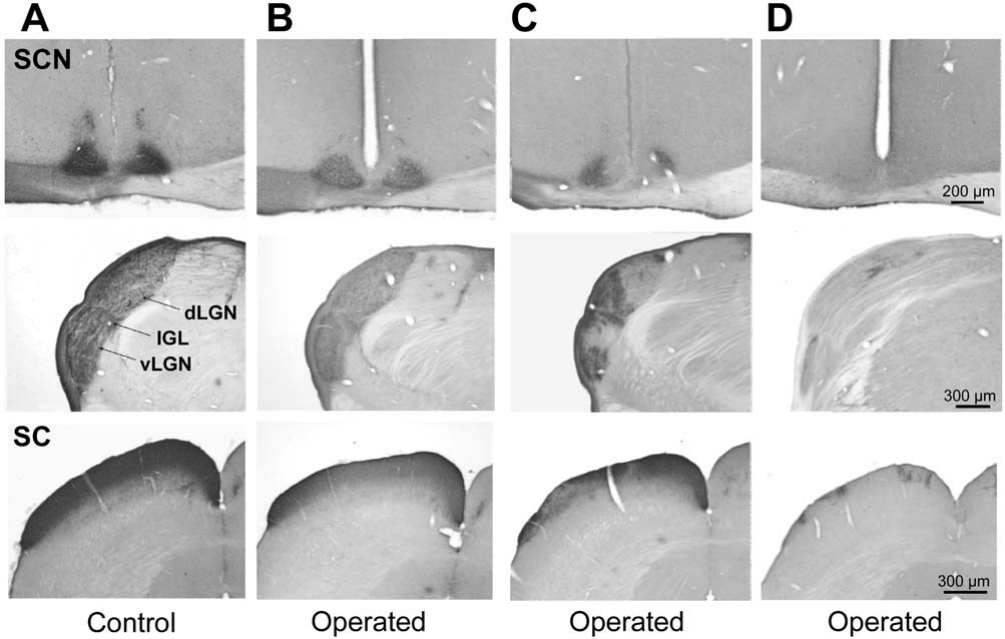
RGC axonal projections to the brain from the eye of a control animal (A) and from the operated eye of animals with monocular induced glaucoma (B–D) following injection with anterograde tracer (CTb). (A) Retinal projections to the brain from a control eye illustrating the normal pattern of dense fiber innervation in the different visual structures. Several cases of experimental glaucoma are illustrated to show the variation of RGC fiber loss in the brain that are (B) reduced but conserve an even distribution of retinal fibers in each visual structure, (C) reduced and show an irregular patchy distribution or (D) are almost completely abolished except for a few small pathes of label. Only contralateral sides of the dLGN and SC are shown. SCN, suprachiasmatic nucleus; dLGN, dorsal lateral geniculate nucleus; vLGN) ventral lateral geniculate nucleus; IGL, intergeniculate leaflet; SC, superior colliculus.

While the double fluorescent tracing method allows a direct qualitative assessment of the topographical distributions of RGC fibers from glaucomatous and control eyes, the use of different fluorescent filters precludes a precise quantitative comparison. For this reason we used optical density analysis of digitalized images from brain sections that were processed for DAB immunocytochemistry [34], [36]. All control rats (8/8) showed dense retinal projections to the all target structures (Fig. 3A). Retinal projections were predominantly contralateral for structures such as the dLGN (94.3±0.9%), the vLGN (89.1±1.7%), the PRT (92.8±1.2%) and the SC (99.8±0.1%). In the SCN and intergeniculate leaflet (IGL) projections were more bilaterally balanced with respectively 65.1±1.6% and 67.4±2.8% of the retinal fibers on the contralateral side. In the SCN, the density of the projection was greater ventro-laterally. For the dLGN, vLGN, IGL and PRT, retinal projections on the contralateral side are relatively homogeneous and occupy virtually the entire extent of each nucleus. Ipsilaterally retinal axons in these structures are sparse and typically distributed in patches, for example in the dorsomedial dLGN and the rostral SC.

Chronically elevated IOP alters these topographical distributions and causes a reduction of the RGC projections in brain structures, but these features vary depending on the individual case. Some laser operated animals showed a uniform reduction of retinal projections in all structures, although the fiber distribution appeared almost normal within the nuclei (Fig. 3B). Other animals displayed a patchy reduction of the density of retinal projections in specific sub-regions of the different brain structures (Fig 3C). In this case, the pattern of the projections onto the contralateral SC suggested that the retinal quadrants most affected were located ventral and lateral to the optic disc. Finally, other individuals with laser-induced glaucoma had severe reductions of retinal projections to all structures, although some sparse patches were observed in the contralateral SC (Fig. 3D). Based on the fiber pattern distribution in the contralateral SC, two of these rats conserved sparse projections from the dorso-temporal region of the retina and the other rat from dispersed retinal regions.

**Figure 4:**
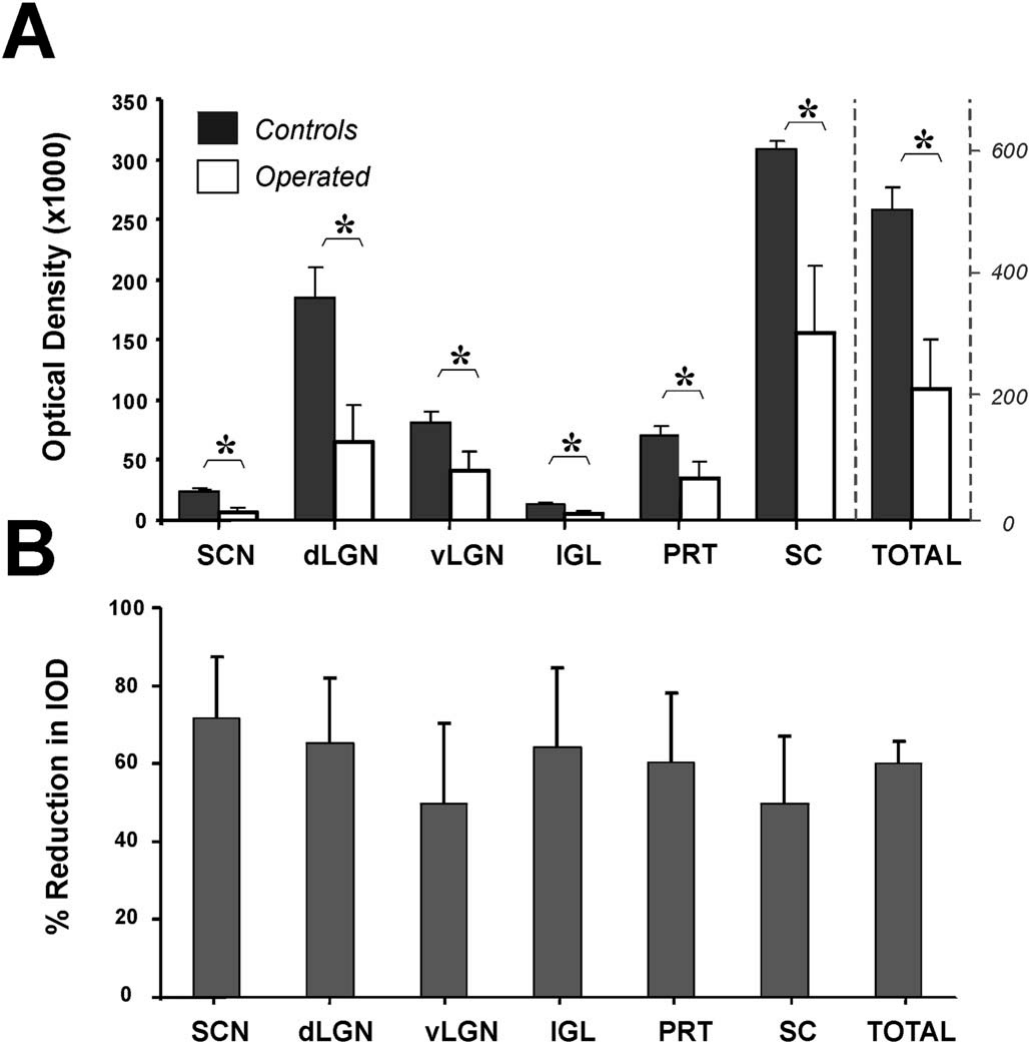
Quantification of the RGC axon terminal projections to all visual structures in control and glaucomatous animals. Structures analyzed in control animals (n = 8, black) and operated animals (n = 6, white) include the suprachiasmatic nucleus (SCN), dorsal lateral geniculate nucleus (dLGN), ventral lateral geniculate nucleus (vLGN), intergeniculate leaflet (IGL), pretectum (PRT) and superior colliculus (SC). For each structure, the ipsi and contralateral densities are summed and represent the integral optical density (IOD) for all the sections obtained from the entire structure. The scale on the right corresponds to values for the total summed density of RGC projections for all structures). The difference in IOD was significant for all structures and for the total amount of projections. (Mann Whitney; * p<0.05). B shows the percent reduction of the IOD value of retinal fiber projections in visual structures of laser-operated eyes compared to the mean IOD value of the projections from unoperated control eyes. Percent reductions range from ∼50–70% depending on the structure. Errors bars in A and B are S.E.M.

The quantitative analysis of the mean densities of retinal projections to different brain structures for operated and control groups and the relative percent reduction (compared to the average values for the controls) are illustrated in Figure 4A–B. Two operated animals omitted from the analysis due to an unacceptable number of missing sections showed qualitative alterations similar to the other individuals. Despite the high degree of variability in the glaucomatous rats, the reduction in retinal fiber density in different structures ranged from 49.7±12.6 % (vLGN) to 71.7±9.4% (SCN) and was significant for each structure (Mann Whitney; p<0.05) and for the total density summed for all the structures (60.1±6.9%, Mann-Whitney, p<0.05; Fig 4B).

### Light entrainment of locomotor activity is altered in glaucoma

**Figure 5:**
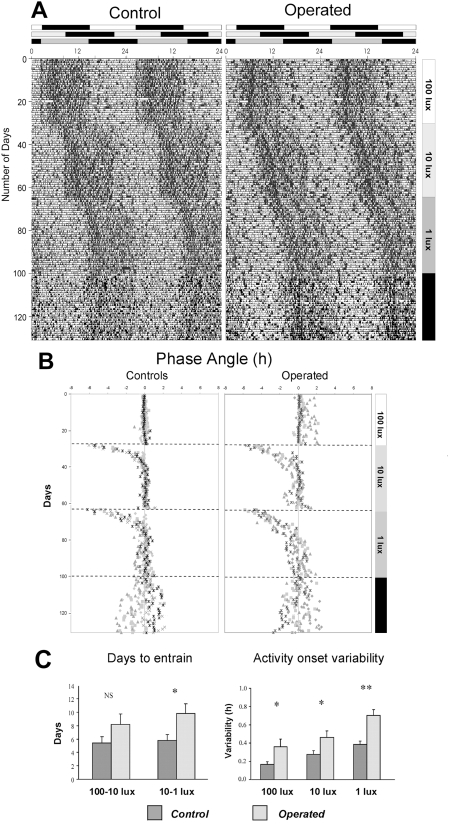
Representative double plot actograms and phase angle plots for a control rat and a rat with experimentally induced binocular glaucoma. Animals are first entrained under a 12:12 light:dark (LD) cycle at 100 lux light (actograms shown in A). After 26 days, the LD cycle was shifted 6 hrs (delay) and the light level was decreased to 10 lux. After 35 days, the light LD cycle was again shifted by 6 hrs and the light level decreased to 1 lux (45 days). Animals were then released into constant darkness to assess whether the animals were entrained to the previous LD cycle. The three successive 12L:12D light cycles (from 100-10-1 lux) are shown above the actograms and the days corresponding to the lux levels of the light phase are indicated on the right. The black bars indicate constant darkness. Although both groups of animals entrained to each of the shifted LD cycles, glaucomatous rats show a greater variability in locomotor activity onsets with respect to the beginning of the dark phase. Some glaucomatous rats also show variability in activity offsets at lights on and components of activity drift during the dark phase. This is illustrated in B for the phase angle plots of the activity onsets of individual control rats (n = 5, left) and rats with binocular glaucoma (n = 5, right). (C) Quantification of the number of days necessary to entrain to a new light-dark cycle (left) and quantification of the activity onset variability with respect to the beginning of the dark phase (right) for both groups. Onset variability was calculated from the last 15 days of each LD cycle, when all animals displayed stable entrainment. (t-test, * p<0.05; ** p< = 0.005).

We then assayed the ability of rats with binocular induced glaucoma to entrain their daily locomotor activity to successive 6 hr delays of LD cycles each coupled with 1 log unit decreases in light levels (100, 10 and 1 lux). Measures of IOP in both glaucomatous eyes showed increases comparable to those recorded for the monocularly operated animals, ranging from 16.1±1.14 mm Hg before surgery to 31.0±1.90 mm Hg following the surgical procedures. Locomotor activity rhythms (double plotted actograms) are shown for a control and operated rat with binocular experimental glaucoma (Fig. 5A). Both control and operated rats were capable of entrainment to the LD cycles at all light levels. However, the animals with binocularly induced experimental glaucoma displayed a behavioral pattern that was characterized by a delay to entrain to the new LD cycle and a high degree of variability for activity onsets in relation to the beginning of the dark phase.

This is illustrated more clearly in the group analysis in Figure 5B where the phase angles of individual activity onsets are shown. The results indicate that the glaucomatous rats require on the average more time to entrain to the new LD cycle but this was only significant when the light level was lowered to 1 lux (control = 5.8±0.86 days, operated = 9.8±1.50 days, t-test, p<0.05; Fig. 5C). The histograms in Figure 5C also show that in comparison to controls, rats with binocular elevated IOP are unable to precisely synchronize their activity to the LD cycle at all light levels, expressed as a significantly greater variability in activity onsets with respect to the beginning of the dark phase (100 lux: control = 0.17±0.02 hrs; operated = 0.36±0.08 hrs, t-test, p<0.05; 10 lux: control = 0.23±0.03 hrs; operated = 0.46±0.10 hrs, t-test, p<0.05; 1 lux: control 0.35±0.02 hrs; operated = 0.64±0.06 hrs t-test, p<0.005).

At the end of the experiment, rats were released in constant darkness to assess whether the animals were entrained to the previous LD cycle at 1 lux light level or if there was a masking component (Fig. 5A–B). Both the control and operated rats were entrained prior to release in constant darkness since the onset of free-running activity extrapolated back to the first day in darkness started to derive from the previous onset point. There was no difference between the two groups in their endogenous free-running periods (control 24.09±0.09 hrs; operated = 24.17±0.71 hrs; Mann-Whitney, p = 0.421) or in the variability of activity onsets (t-test: p = 0.533). This suggests that the onset variability of operated animals in LD is related to an alteration of light input in the glaucomatous rats rather than of circadian clock function itself.

### Alteration of retinal opsin mRNAs and ganglion cell markers in glaucoma

**Figure 6:**
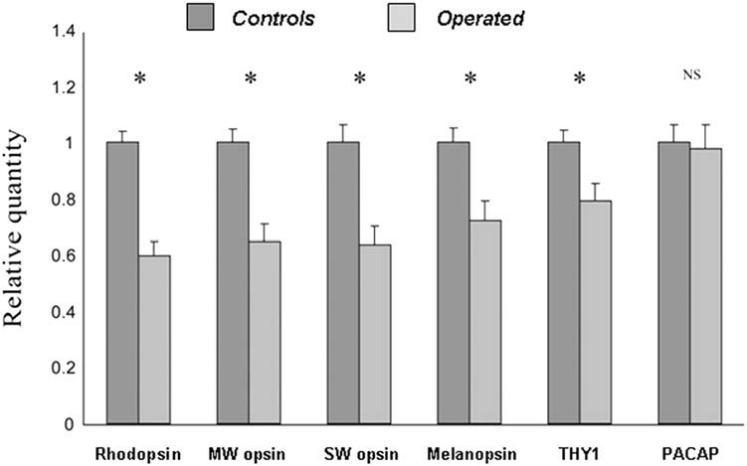
Quantification of mRNA expression of retinal opsins, Thy1 and PACAP. Short wavelength cone (SW), mid-wavelength cone (MW) opsins, rhodopsin and melanopsin mRNA expression are all significantly reduced in experimental glaucoma. The specific RGC marker Thy1 was also significantly reduced whereas PACAP expression is unchanged (t-test, * p<0.05; ** p<0.005.) Both of the retinas from each of the binocularly operated (n = 5) and control rats (n = 5) were used.

Finally, real time quantitative PCR was used to evaluate the expression of different retinal opsins as well as the expression of a specific ganglion cell marker (Thy1) and pituitary adenylate cyclase peptide (PACAP) a neurotransmitter co-expressed in melanopsin RGCs (Fig. 6). As expected, mRNA of melanopsin and Thy1 are significantly decreased in eyes with raised IOP (t-test, p<0.01). In contrast, PACAP mRNA expression remains unchanged (t-test, p = 0.831). However, an unexpected finding was that all opsin mRNAs from the outer retina (SW, MW opsins and rhodopsin) were significantly under expressed in case of experimental glaucoma (t-test, p<0.001).

## Discussion

Previous studies of animal models of experimental glaucoma secondary to chronically elevated IOP have demonstrated significant RGC loss ranging from 30% to 90% depending on the method employed, time course and experimental model [14], [33], [41]–[43]. The assay of RGC degeneration in the retina is a gold standard for assessing the consequences of experimental glaucoma, but in the present study we focused on the loss of central RGC fiber projections in order to gain a comprehensive view of the quantitative and qualitative alterations in the target structures that process visual and non-visual information in the brain. To our knowledge, only two (contradictory) studies have examined the consequences of ocular hypertension on the alteration of RGC fiber projections. One HRP-tracing study in the rat SC [44] claimed a complete lack of anterograde transport, while the second study found 20–90% losses of retinal projections in the magno- and parvocellular layers of the monkey dLGN (silver grain counts; [45]).

Anterograde tracing in the hypertensive rat model indicates that experimental glaucoma induces an overall reduction of the retinal projections to the brain (∼60%) while the same analysis following severe degeneration of outer retinal photoreceptors reveals no effects [37]. The reduction is particularly significant for the SCN, the main target of an almost exclusive innervation from melanopsin RGCs [26] that shows a mean reduction of ∼71%. The dLGN; IGL and PRT show mean reductions ranging from ∼60–65% while the vLGN and SC show a reduction of roughly 50%. This suggests that melanopsin RGCs are equally susceptible to degeneration as other RGCs in raised IOP, consistent with the 65–80% reduction of melanopsin RGCs in the study by Jakobs et al. [14]. These two results contrast with a third report describing a resistance of the melanopsin cell population to IOP induced injury [32]. This discrepancy may arise from the fact that the latter study used a small sample size, short survival periods (<14 days) and different methods to quantify the reduction in melanopsin RGCs and in the total RGC population.

A consistent feature of the reduction of RGC projections, despite the reliable increase of IOP induced by the laser surgery, was the variability observed between individual animals in both the extent and the topographical pattern of loss of RGC fibers. The variation of RGC, optic nerve fiber and visual field loss following increased IOP is a characteristic feature in both experimental and human glaucoma [4]. For example, in mouse models of hereditary increased IOP, 28% of the mice show little or no indication of glaucomatous damage while 66% showed severe damage, including differences between the left and right eyes in a single individual [14]. Furthermore, the topographical pattern of degeneration across the retinal surface can show considerable variation [14], [46]. The nature of the mechanistic link between high intraocular pressure and loss of retinal ganglion cells is still not fully understood and although raised IOP is clearly an important risk factor, patients with ocular hypertension do not invariably progress to clinical glaucoma and RGC degeneration, even over long-term periods [2], [4], [47]–[49].

The reduction in mRNA of melanopsin and of the ganglion cell marker Thy1 also confirms the overall decreases of the RGC population and of melanopsin RGCs. In contrast, PACAP, which is co-expressed in melanopsin RGCs [50] remains unchanged. However, it is unclear whether PACAP is also expressed in other retinal neurons and a decrease in melanopsin mRNA without a concomitant decrease in PACAP is observed in rats treated with N-methyl-N-nitrosourea (MNU), a pharmacological agent causing photoreceptor apoptosis [51].

An unexpected but significant finding is that mRNA opsin expression from outer retinal photoreceptors (MW cones, SW cones, rods) were all found to be under expressed. Although the question of whether other retinal cell types are altered in glaucoma is still a matter of debate, Jakobs et al. [14] using well-characterized cell markers for specific amacrine and bipolar cells coupled with morphological analysis of soma and dendritic architecture argue that glaucoma affects exclusively the RGCs. Most early histopathological studies reported no photoreceptor loss in human eyes with primary open angle glaucoma [52] or in a monkey model of glaucoma [53]. More recent data describes minor abnormalities without cell loss in the outer retina, including swollen photoreceptors in both the human disease and in the monkey [54]. Furthermore, multifocal ERG studies have shown that the outer retina is functionally affected in experimental glaucoma [55]–[56]. Finally, a recent investigation [57] using *in situ* hybridization showed a reduction in the expression of MW/LW and SW cone opsin mRNAs in monkeys with chronic ocular hypertension, consistent with our results. In contrast no changes in the rod opsin mRNA level were observed. Taken together, these data suggest that although the outer retina is not anatomically altered in experimental or human glaucoma, cone and rod opsin mRNAs are under expressed and may be functionally impaired. The reduced level of mRNA opsins may result directly from the chronic increase in IOP or, alternatively, may be an indirect effect related to the partial loss of melanopsin RGCs and a subsequent disruption of the circadian regulation of retinal physiology and outer retinal photoreceptor processes [58]. If the retinal clock is deregulated, this may have potentially important consequences on gene cycling, photopigment regeneration and retinal function [59] in severe glaucomas.

The reduction of melanopsin RGCs and their innervation of the SCN impacts on the ability of glaucomatous rats to entrain to light. We used an entrainment paradigm since this assay is reported to be more sensitive for detecting entrainment deficits compared to single light-pulse phase shifts in both animals [60] and humans [61] and is more relevant to real-life conditions that human patients are exposed to. Rats with binocular hypertension require more time to re-entrain to a shifted LD cycle at low light levels compared to control rats and display greater variability in the activity onsets. Mice invalidated for melanopsin show a deficit in their ability to entrain at low light levels, to phase shift to light [20], [21], [26], [38], have impaired masking responses to light [22] and show severely reduced photic responsiveness in the SCN [28]. However, the total loss of melanopsin photopigment in *Opn_4_* ^−/−^ mice is not equivalent to the situation in glaucomatous rats (or human glaucoma), where a variable proportion of melanopsin RGCs (and their rod/cone inputs) are absent. Our results are similar to the anatomical and behavioral alterations recently reported using a targeted saporin-based immunotoxic technique that results in a partial ablation of melanopsin RGCs [62].

In humans, it has been reported that patients with different degrees of blindness resulting from various ocular pathologies show sleep disturbances and abnormal circadian rhythms [29]–[31]. These disturbances were attributed to a lack of light input to the circadian clock and are correlated with the degree of loss of light perception. Patients with the lowest level of conscious light perception show the greatest degree of sleep impairments. In the study by Tabandeh et al. [31] the patient group with optic nerve disease/glaucoma showed a higher likelihood of sleep disorders, although this group contained individuals with light perception and others with no light perception. Glaucoma patients have been shown to exhibit relative afferent pupillary defects in early stages [63]–[65] and a prevalence of sleep disorders in later stages [66]–[67]. In our laboratory, preliminary studies of patients with severe open angle bilateral glaucoma show a high variability in both the normal time to bed and in the dim-light melatonin onset [68] which we speculate represent the main manifestations of circadian disorders in this group.

Here, we provide evidence that chronic increase IOP in a rodent model of glaucoma leads to a decrease in the melanopsin RGC axonal innervation of the SCN and an alteration in the photic response to light. The alteration of entrainment of locomotor activity in this model suggests that patients with severe bilateral glaucoma may show an increased propensity for chronobiological disturbances. Our data and that of previous studies support the idea that RGC loss due to glaucoma affects both visual and non-visual functions. Concerns for health and quality of life for patients with glaucoma should thus not be limited to the detection and prevention of visual impairments but should also consider the potential impacts on altered circadian entrainment and sleep disturbances.
